# An endogenous ‘non-specific’ protein detected by a His-tag antibody is human transcription regulator YY1

**DOI:** 10.1016/j.dib.2014.12.002

**Published:** 2014-12-31

**Authors:** Niaz Mahmood, Jiuyong Xie

**Affiliations:** a Department of Biochemistry & Medical Genetics, College of Medicine, Faculty of Health Sciences, University of Manitoba, Winnipeg, MB, Canada R3E 0J9; b Physiology & Pathophysiology, College of Medicine, Faculty of Health Sciences, University of Manitoba, Winnipeg, MB, Canada R3E 0J9

**Keywords:** His-tag antibody, Yin and Yang 1 (YY1), Histidine-rich proteins, HeLa, HEK293T, ‘Non-specific’ band

## Abstract

Histidine-tags have been used for a wide variety of experiments including protein purification, Western blots, immunoprecipitation and immunohistochemistry. In our previous studies, we have repeatedly detected a ‘non-specific’ endogenous protein of about 60 kD in Western blots of protein lysates from HEK293T or HeLa cells using the anti-His-tag antibody (His-probe (H3), catalogue #, SC-8036, Santa Cruz Biotech. Co.) (Yu et al., J. Biol. Chem. 284 (2009) 1505–1513). Here we have immunoprecipitated the protein from HeLa nuclear extracts using the anti-His-tag antibody, excised the 60 kD band and subjected it to LC–MS/MS (Fig. 1). The deduced sequences of two peptides of the protein match the human transcriptional regulator YY1 (Yin and Yang 1, UniProt ID, P25490, Fig. 2), which contains 11 histidine residues in a stretch (from amino acid 70 to 80) at its NH_2_-terminal region without known functions (Lee et al., Nucleic Acids Res. 23 (1995) 925–931; Bushmeyer et al., J. Biol. Chem. 270 (1995) 30213–30220). Since genes encoding other Histidine-repeat proteins also exist in the genome (Salichs et al., PLoS Genet. 5 (2009) e1000397), it is possible that YY1 might not be the only endogenous protein that could be expressed and recognized by the antibody in different sources of samples in future experiments. The presence of various endogenous histidine-repeat proteins suggests that data from experiments particularly immunostaining using His-tag antibodies need to be interpreted with caution. This might also be useful to the broader scientific community by providing an example for the interpretation of ‘non-specific’ bands in Western blots.

**Specifications table**

**Table:** 

Subject area	*Biochemistry*
More specific subject area	*Proteomics*
Type of data	*Text and figure*
How data was acquired	*Immunoprecipitation, LC–MS/MS*
Data format	*Analyzed*
Experimental factors	*Human cell lines (HeLa and HEK293T)*
Experimental features	*Immunoprecipitation using an antibody against His-tag repeatedly detected a non-specific band which was subject to mass spectrometry after immunoprecipitation.*
Data source location	*N/A*
Data accessibility	*Within this article*

## Value of the data

• The data will let other researchers know the identity of the ‘non-specific’ protein band in Western blots detected by the anti-His-tag antibody [His-probe (H3), catalogue #, SC-8036] in two of the most widely used human cell lines HeLa and HEK293T.
• Data using His-tag antibodies, particularly for immunohistochemistry, should be interpreted with caution by taking into consideration of the endogenous antigens.
• Detectable changes in this band in future studies would suggest to one that the transcription regulator is perhaps altered.
• This provides an example for the interpretation of ‘non-specific’ bands in Western blots.

## 1 Experimental design, materials and methods

**Fig. 1:**
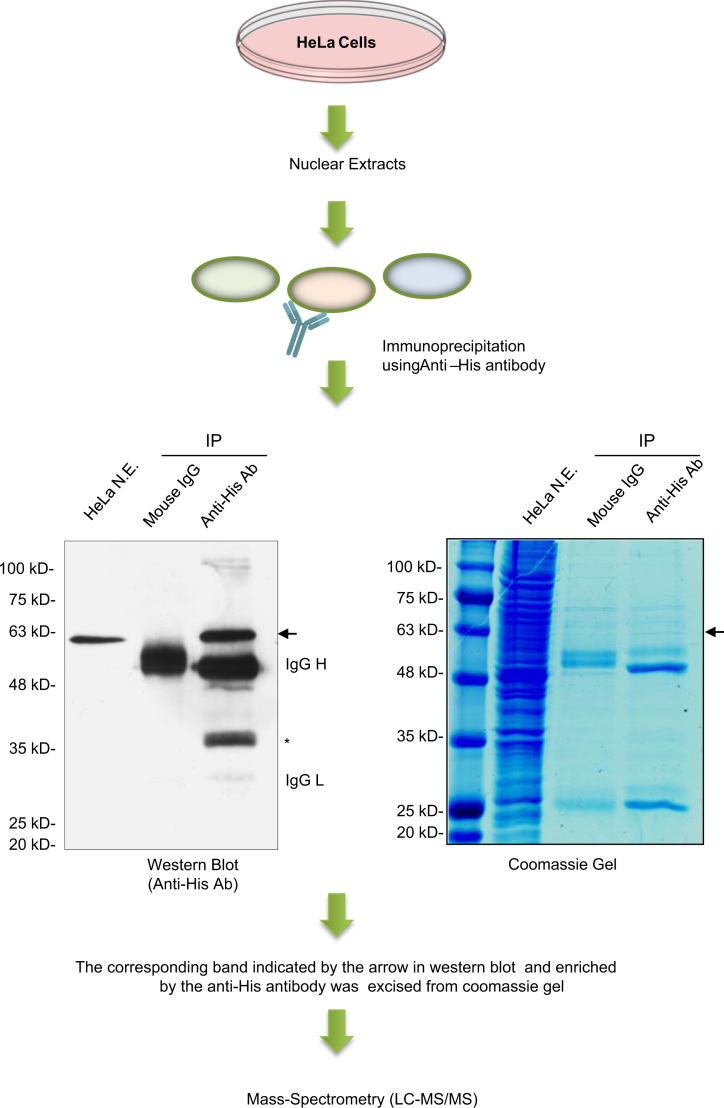
Flow chart of the methods used for acquisition of the data. Protein band indicated by the arrow in the representative images was excised from the coomassie gel for mass spectrometry. *: a band detected in the IP product but not in the nuclear extract sample in Western blot.

Fig. 1 shows the flow chart of the methods used to acquire the data. Cell culture, nuclear extract preparation and immunoprecipitation were as described previously [1]. The immunoprecipitated proteins were run onto a SDS-polyacrylamide gel and the ‘non-specific’ 60 kD band was cut with a clean blade and sent for LC–MS/MS analysis in the Southern Alberta Mass Spectrometry (SAMS) Centre. The MS/MS ions data was searched against the human proteins in the SwissProt database using the ‘MS/MS ions’ search at the Mascot server (http://www.matrixscience.com/).

## Conflict of interest

The authors declare no conflicts of interest.

## Figures and Tables

**Fig. 2:**
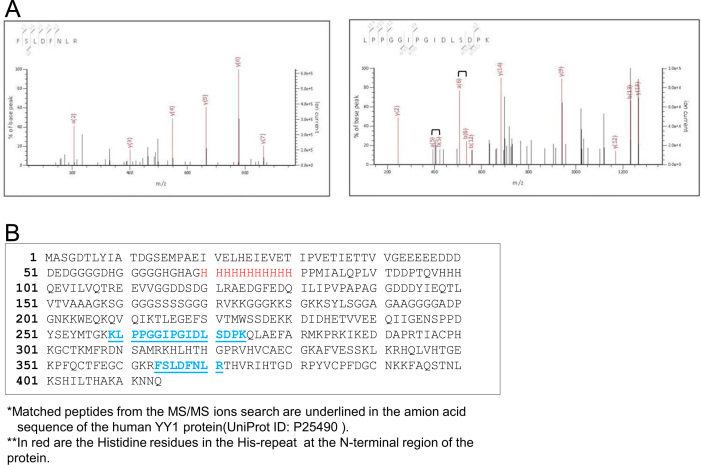
Results from the LC–MS/MS of the 60 kD protein. (A) MS/MS spectra of the peptides and amino acid sequences deduced from the spectra. The peaks used for scoring are highlighted in color. Indicated above each peak is the corresponding amino acid deduced from the a-, b- or y-type ions. MS/MS ion search at the Mascot server showed that the two peptides match with YY1 significantly (*p*<0.05) with a protein score of 41 compared to the random event (<30). (B) The region matched by the peptides from LC–MS/MS (underlined, in blue) and the histidine-repeat (in red) are shown in this full-length amino acid sequence of the human YY1 protein (UniProt ID, P25490) [2-4].
